# Effect of pigmentation intensity of trabecular meshwork cells on mechanisms of micropulse laser trabeculoplasty

**DOI:** 10.1038/s41598-022-14637-5

**Published:** 2022-06-22

**Authors:** Shota Shimizu, Megumi Honjo, Koichiro Sugimoto, Michiaki Okamoto, Makoto Aihara

**Affiliations:** 1 Department of Ophthalmology, Graduate School of Medicine, The University of Tokyo, 7-3-1, Hongo, Bunkyo-ku, Tokyo 113-8655 Japan; 2 Tomey Corporation, Nagoya, Japan

**Keywords:** Ocular hypertension, Glaucoma, Biochemistry, Medical research

## Abstract

The intraocular pressure (IOP)-lowering mechanisms of micropulse laser trabeculoplasty (MLT) remain unclear. The present study was performed to investigate the mechanism of action of MLT, and to determine whether the pigmentation intensity of trabecular meshwork (TM) cells is associated with the treatment effects. Primary human TM cells were exposed to melanin granules to artificially introduce different levels of pigmentation. Micropulse (MP) laser irradiation was performed, and interleukin (IL)-1α/β, matrix metalloproteinases (MMPs), tissue inhibitor of metalloproteinases (TIMPs), and extracellular matrix (ECM) protein expression were evaluated by RT-qPCR and immunocytochemistry. IL-1α/β and MMP-1, -3, and -9 mRNA expression were significantly upregulated at 4 and 24 h after MP laser irradiation, respectively, but there were no significant changes in TIMP expression. The extent of these upregulation was greater in cells with strong pigmentation intensity. Protein expressions of fibronectin and collagen I were significantly decreased in cells with strong staining intensity. These results suggested that MP laser irradiation alter the MMP/TIMP ratio and enhance ECM turnover, resulting in increased outflow of aqueous humor. The pigmentation intensity of the TM tissues may affect the treatment efficacy of MLT, because TM cells with strong staining intensity showed a significantly enhanced response to MP laser irradiation.

## Introduction

Glaucoma is characterized by visual field loss associated with damage to the optic nerve, and it is a leading cause of irreversible blindness. Elevated intraocular pressure (IOP) is an important risk factor for glaucoma, and IOP reduction is the only proven treatment method^1,2^. IOP homeostasis is maintained by the balance between secretion of aqueous humor by the ciliary body and its drainage through the trabecular meshwork (TM) and uveoscleral outflow pathways. Increased resistance of aqueous outflow through the TM has been reported in open-angle glaucoma (OAG), which is the most common form of glaucoma^1,3^. The standard first-line treatment for OAG and ocular hypertension (OH) is drug treatment with daily eye drops to lower IOP, but poor patient adherence has been a problem^2,4,5^. Laser trabeculoplasty has attracted renewed attention as a primary or adjunctive therapy for lowering IOP that does not require patient adherence.

Argon laser trabeculoplasty (ALT) has been performed since the 1970s, and its IOP-lowering effect was reported to be mediated by an increase in TM outflow^6^. Selective laser trabeculoplasty (SLT) was introduced in the late 1990s as a new laser treatment modality with a different mechanism of action from ALT^7,8^. SLT selectively targets TM cells and delivers much less than the total energy used by typical ALT, without causing collateral damage to adjacent non-pigmented cells or structures, or thermal coagulative effects, in contrast to ALT^9–11^. SLT has been shown to be as effective as drugs that lower IOP, and to be more cost-effective than drugs or surgery. The number of trabeculoplasties has increased significantly over the past decade^12,13^. Micropulse laser trabeculoplasty (MLT) has recently been developed to decrease TM tissue damage^10,11,14^. In micropulse (MP) laser irradiation, repeated short pulses of irradiation with intervening breaks allows cooling of the tissue (via thermal diffusion between pulses), which minimizes structural destruction of the irradiated area and secondary thermal damage. A transient postoperative IOP increase is often observed after SLT, but the incidence of this transient IOP elevation was reported to be lower in MLT^15–17^. Previous studies suggested that MLT is comparable to SLT in terms of efficacy and effectiveness, and may have a better safety profile. However, the mechanism of action MLT remains unclear^17,18^.

Studies using cultured TM cells and explanted tissue cultures showed that laser irradiation upregulates the expression of matrix metalloproteinases (MMPs)^19–22^. MMPs are a large family of enzymes that degrade the constituent proteins of the extracellular matrix (ECM), and it has been suggested that the one of the mechanisms of IOP reduction by laser trabeculoplasty may involve enhancement of ECM turnover via upregulation of MMPs and alteration of the MMP/tissue inhibitor of metalloproteinases (TIMPs) ratio^22–27^. In addition, the pigmentation intensity of the TM has been suggested to be correlated with the treatment effect of laser trabeculoplasty^17,28,29^. Several clinical studies reported a positive correlation between the pigmentation intensity of the TM and therapeutic effect of SLT, and significant IOP reduction was observed only in the strong TM pigmentation group^29^. In some other studies, however, pigmentation intensity of the anterior angle of the eye was not related to the effect of SLT treatment^17,28^. There have been insufficient clinical studies, and no in vitro studies, regarding this issue. The pigment intensity of the TM is influenced by a variety of factors, including race, age, and type of glaucoma. In particular, the pigment intensity of the TM is high in pigment dispersion glaucoma and pseudoexfoliation glaucoma, which is thought to contribute to increased outflow resistance and elevated intraocular pressure^30,31^. Therefore, the relationship between pigmentation intensity and laser response should be investigated even in MLT, a relatively new laser trabeculoplasty. However, the correlation between pigmentation intensity and its response to micro pulse laser irradiation has not been reported.

Here, we investigated whether MMPs are involved in the mechanism of action of MLT treatment, and whether the pigmentation intensity of the TM has an effect. Our results showed that MP laser irradiation upregulates the expression of cytokines and MMPs in cultured TM cells while controlling for pigmentation intensity. Furthermore, this is the first study to show that strong pigmentation of TM cells contributes to the response to MP laser irradiation, and may also play a role in lowering IOP.

## Methods

### Preparation of melanin suspension

Melanin suspension was prepared with reference to previous reports^27,32^. Briefly, 10 mg of melanin granules (Sigma-Aldrich, St. Louis, MO, USA) were suspended in 10 mL of purified water. For reduction of particle size, the preparations were sonicated five times at 10-s intervals, for 30 s each time. To exclude large particles, the suspension was centrifuged at 300 × *g* for 2 min and the precipitate was removed. The suspension was left to stand for 24 h, and the supernatant was removed to exclude small particles. These procedures ensured size uniformity of the melanin granules. Melanin suspensions at concentrations of 0.2, 0.1, and 0.05 mg/mL were prepared by resuspending the precipitates in fresh medium. The concentration of the melanin suspension was controlled by constructing a calibration curve based on the absorbance at 620 nm (A_620_).

### Cell preparation and artificial pigmentation

In all experiments, primary human TM (hTM) cells (Cat #6590, ScienCell Research Laboratories, San Diego, CA, USA) were used between passages 4 and 6. hTM cells were cultured in Dulbecco’s modified Eagle’s Medium/Nutrient Mixture F-12 medium supplemented with 10% fetal bovine serum (ScienCell Research Laboratories) and 1% antibiotic–antimycotic solution (100×; Sigma-Aldrich) at 37 °C in an atmosphere of 5% CO_2_. The identity of the cells was confirmed by dexamethasone-induced upregulation of myocilin (MYOC) and immunocytochemistry of AQP1, TIMP3, vimentin and desmin according to previous reports^33^. Exposure to 100 or 500 nM dexamethasone (Sigma-Aldrich) was confirmed to enhance MYOC expression compared to vehicle (DMSO)-treated cells (Supplementary Fig. S1). Furthermore, it was confirmed positive for AQP1, TIMP3, and vimentin, and negative for desmin in immunocytochemistry (Supplementary Fig. S2). Non-pigmented hTM cells were seeded in 24-well plates at a concentration of 6.0 × 10^4^ cells/cm^2^ and cultured for 24 h. After washing with phosphate-buffered saline (PBS), these cells were exposed to three concentrations of melanin granules for 24 h to artificially introduce different levels of pigmentation (weak, moderate, or strong). Melanin granules not taken up by the cells were removed by washing twice with PBS. Fresh medium was added (600 μL/well) for laser irradiation. For measurement of melanin in the prepared pigmented hTM cells, cells were lysed in RIPA buffer, and the melanin concentration was determined by measuring the A_620_.

### Micropulse laser treatment

MP laser irradiation was performed with a IQ577^®^ laser (Iridex Corp., Mountain View, CA, USA) at 700–1500 mW (577 nm, 15% duty cycle, duration of 0.2 s, diameter of 300 μm). To allow vertical irradiation onto the cultured cells, the main unit was rotated 90° and fixed to a vertical metal arm attached to a horizontal movable stage. A TxCell™ Scanning Laser Delivery System (Iridex Corp.) was used for accurate and efficient spot placement. The plates were irradiated with a total of 600 shots per well, calculated to cover approximately 50% of the total surface area of each well. The laser power was corrected according to the irradiation conditions by measuring the actual output power using a power meter (PM100D; Thorlabs Inc., Newton, NJ, USA).

The amount of energy absorbed by the pigmented cells due to laser irradiation was evaluated using a power meter, as follows. Three groups of hTM cells with different pigmentation intensities were irradiated with a 700–2000 mW MP laser, and the power of transmitted light was measured. Non-pigmented cells were used as controls. The energy absorbed by the pigmented cells was calculated by averaging the measurements obtained for cells irradiated at five different locations independently for each condition, and subtracting the measurements of non-pigmented cells.

### Observations of cell morphology and metabolic activity

The damage to the cells after laser irradiation was measured in 24-well plates using a CCK-8 assay kit (Dojindo Laboratories, Kumamoto, Japan), which measures cellular dehydrogenase activity. At the same time, the cells were observed by phase contrast microscopy to check for changes in cell morphology and the presence of floating cells.

### RT-qPCR

Total RNA was extracted from hTM cells at 4 or 24 h after laser irradiation using an AllPrep™ DNA/RNA Mini kit (QIAGEN, Valencia, CA, USA) according to the manufacturer’s protocol. The concentration of total RNA was determined by measuring the absorbance at 260 nm (A_260_) using a spectrophotometer (NanoDrop 2000; Thermo Fisher Scientific, Waltham, MA, USA).

The total RNA was reverse-transcribed into complementary DNA using ReverTra Ace qPCR RT Master Mix with gDNA remover (Toyobo, Osaka, Japan). Quantitative PCR (qPCR) was performed with a Thermal Cycler Dice Realtime System (Takara Bio, Shiga, Japan). The sequences of PCR primers used are presented in Supplementary Table S1. Target gene expression was normalized relative to that of GAPDH mRNA.

### Enzyme-linked immunosorbent assay (ELISA)

Cell culture media were collected at 24 h, 3, and 14 days after MP laser irradiation, and stored at − 80 °C until measurement. The MMP-3 and TIMP-1 secretions were quantified using an MMP-3 Human ELISA Kit (Thermo Fisher Scientific) and Human TIMP-1 ELISA Kit (Thermo Fisher Scientific) according to the manufacturer’s protocols.

### Immunocytochemistry

The cells were cultured on cover glasses in 24 well plates. Immunocytochemistry was performed 1 week after laser irradiation as described previously^34^. For melanin bleaching of pigmented cells, the cells were fixed with 4% paraformaldehyde and then treated with 10% hydrogen peroxide at 4 °C overnight. The primary antibodies were anti-FN (1:500; Santa Cruz Biotechnology, Dallas, TX, USA) and anti-COL1A1 (1:200; Rockland Immunochemicals, Limerick, PA, USA). Alexa Fluor 488- and 594-conjugated secondary antibodies (1:1000) were purchased from Thermo Fisher Scientific. These slides were imaged under a fluorescence microscope (BZ-9000; Keyence, Osaka, Japan). Six images of each well were taken, and the mean fluorescence intensity was calculated using Fiji/ImageJ v1.53 (Java 1.8.0_172)^35^. The measured fluorescence intensity was normalized relative to the number of DAPI-positive cells. The intensity of the laser-irradiated group was calculated relative to the non-irradiated control (taken as 100%). Three wells were used for each condition, and the mean values were calculated.

### Statistical analysis

The results are expressed as the mean ± standard deviation (SD). Differences between two groups were analyzed with Student’s *t* test. Comparisons between more than two groups were performed with Dunnett’s multiple comparisons test using R (version 4.1.2)^36^. All data represent the means of at least three independent experiments. In all analyses, *P* < 0.05 was taken to indicate statistical significance.

## Results

### Measurement of melanin uptake and laser energy absorption by pigmented cells

**Figure 1:**
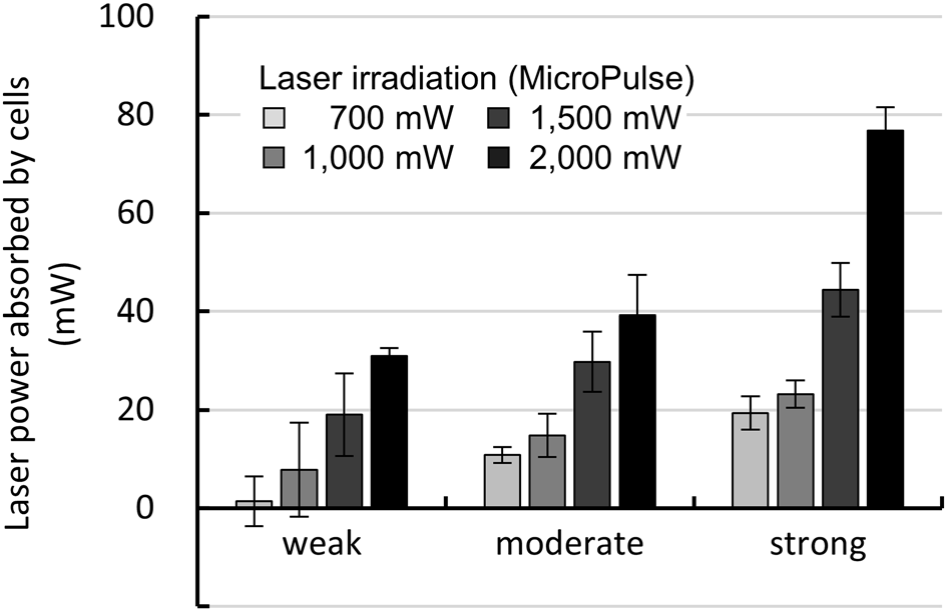
Differences in power absorbed by cells depending on stain intensity and laser irradiation power. The power of the laser transmitted through the cell culture surfaces were measured, and the laser power (mW) absorbed by the cells was calculated using the cells without pigmentation as a blank (n = 5). Data are represented as mean ± standard deviation of the mean.

The amounts of melanin granules per cell corresponding to weak, moderate, and strong pigmentation were 64 ± 1.2, 147 ± 1.7, and 336 ± 1.5 pg/cell, respectively (Table 1). We found that the amount of melanin taken up could be controlled by changing the concentration of the suspension to which the cells were exposed. We also confirmed that the amount of melanin did not change when cells with different pigmentation intensities were exposed to MP laser irradiation (Table 1). Next, we measured the actual energy absorbed by pigmented hTM cells under MP laser irradiation at 700, 1000, 1500, and 2000 mW using a power meter. The energy absorbance was greater with stronger pigmentation intensity and higher irradiation energy (Fig. 1).

**Table 1:** Melanin concentration of artificially pigmented cells (pg/cell).

	Non-laser	700 mW	1000 mW	1500 mW
None	–	–	–	–
Weak	64 ± 1.2	65 ± 2.0	67 ± 2.1	69 ± 1.5
Moderate	147 ± 1.7	141 ± 5.5	147 ± 3.5	143 ± 1.2
Strong	336 ± 1.5	329 ± 5.9	350 ± 3.8	336 ± 3.6

### Effects of MP laser irradiation on cell morphology and metabolic activity

**Figure 2:**
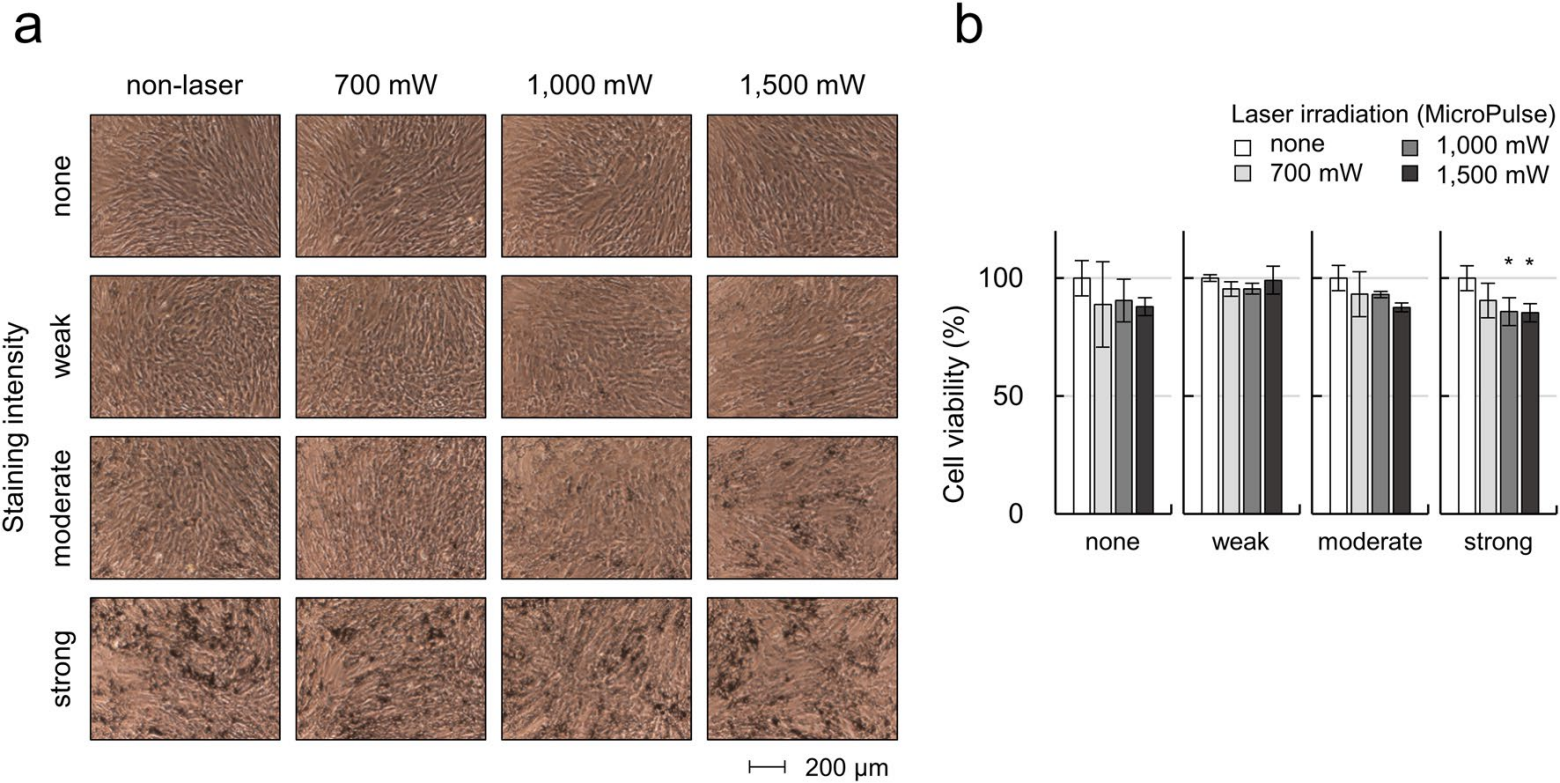
Cell damage caused by MP-laser irradiation. (**a**) Observation of morphological changes and aggregation of the laser spots by the phase-contrast microscopy images after laser irradiation. Scale bars: 200 μm. (**b**) Metabolic activity of pigmented TM cells was measured 4 h after MP-laser irradiation by CCK-8 assay (n = 3). Data are represented as mean ± standard deviation of the mean. *P < 0.05 by Dunnet’s multiple comparisons test.

To determine whether the pigmentation or MP laser irradiation affected cell viability, hTM cells with each pigmentation intensity were treated with MP laser irradiation at 700, 1000, and 1500 mW. No clear morphological changes or aggregation of the laser spots were observed on phase-contrast microscopy after irradiation (Fig. 2a). Furthermore, no suspended or dead cells were found. As shown in Fig. 2b, there were no significant changes in the metabolic activity of hTM cells at the different pigmentation intensities compared to controls, but the metabolic activity of hTM cells with strong pigmentation was reduced by 9.5%, 14.2%, and 14.7% at 4 h after 700, 1000, and 1500 mW MP laser irradiation compared to non-irradiated controls. The reductions following irradiation at 1000 and 1500 mW were statistically significant compared to the non-irradiated controls. There were no significant reductions in metabolic activity in cells with no, weak, and moderate pigmentation. These results suggested that neither pigmentation nor MP laser irradiation significantly altered cell morphology, but that MP laser irradiation tended to decrease the metabolic activity of cells depending on the intensity of pigmentation.

### Evaluation of IL-1α/β mRNA expression levels

**Figure 3:**
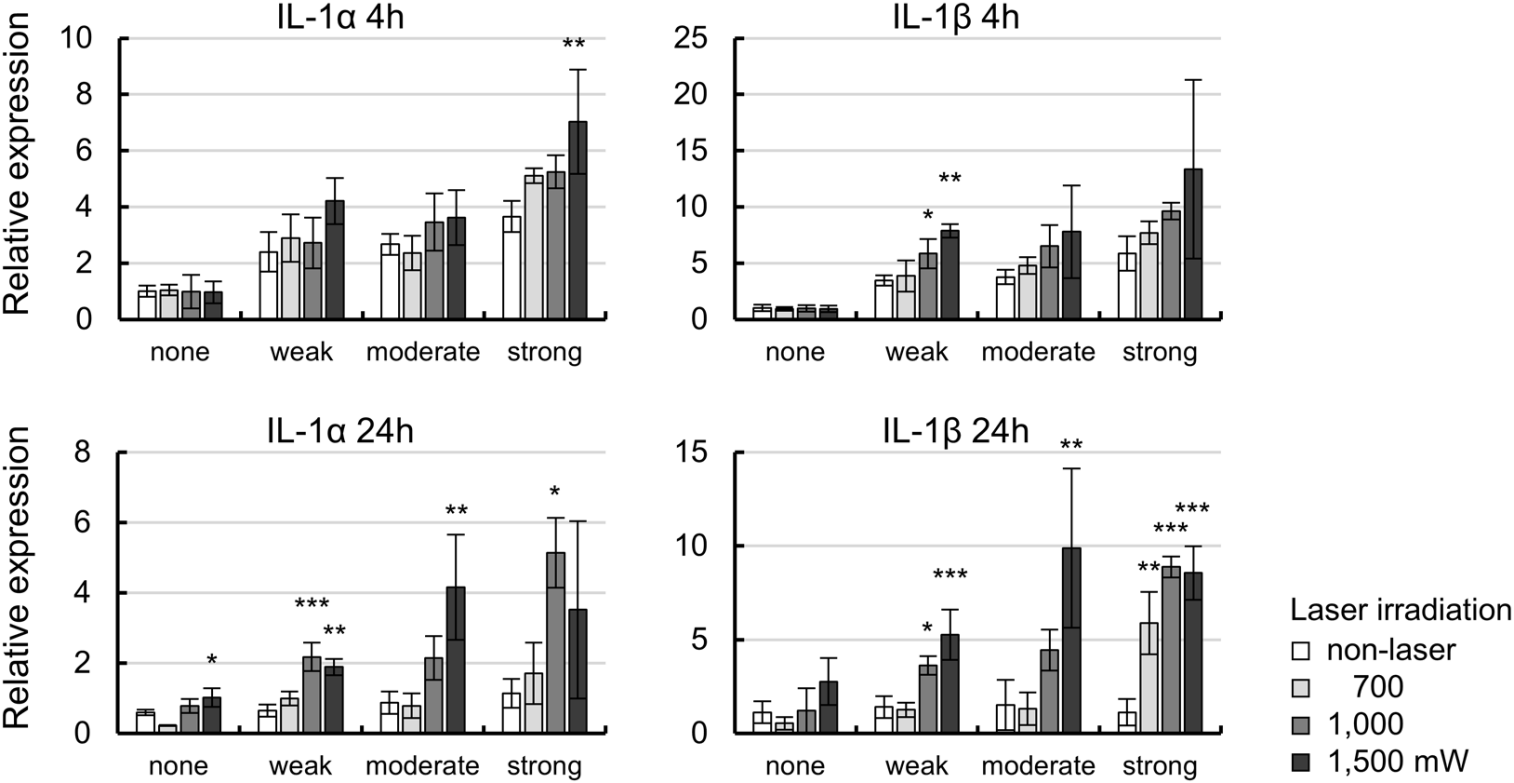
Expression levels of IL-1α/β after 4 or 24 h of MP-laser irradiation of cells with each pigmentation intensity. mRNA levels of IL-1α/β were measured by qPCR (n = 3). Data are represented as mean ± standard deviation of the mean. *P < 0.05, **P < 0.01, ***P < 0.001 by Dunnet’s multiple comparisons test.

To confirm the changes in cytokine expression levels due to laser irradiation, total RNA was extracted from pigmented TM cells at 4 or 24 h after laser irradiation, and subjected to RT-qPCR analysis (Fig. 3). In the pigmented cells without MP laser irradiation, the levels of interleukin (IL)-1α/β mRNA expression were higher than in non-pigmented cells at 4 h (IL-1α: *P* = 0.01, = 0.005, < 0.001, IL-1β: *P* = 0.01, = 0.005, < 0.001, for weak, moderate, strong, respectively by Dunnet’s multiple comparisons test). The levels of IL-1α/β expression were increased compared to non-irradiated controls at 4 h after MP laser irradiation at all each cell pigmentation intensities, but IL-1α was significantly upregulated in cells with strong pigmentation irradiated at a power of 1500 mW (*P* < 0.01, Dunnet’s multiple comparisons test). IL-1β was significantly upregulated in cells with weak pigmentation irradiated at 1000 and 1500 mW (*P* = 0.043, = 0.001 for 1000 and 1500 mW, respectively, Dunnet’s multiple comparisons test). It is possible that IL-1α/β expression was induced by the pigmentation, but MP laser irradiation may also have affected the upregulation of these cytokines. As there were no significant differences according to pigmentation after 4 h, we further compared the effects of MP laser irradiation on cytokine expression after 24 h.

As shown in Fig. 3, the levels of IL-1α/β expression were increased compared to the non-irradiated controls at 24 h after MP laser irradiation in each pigmentation intensity group. Upregulation of IL-1α mRNA level tended to increase according to the laser power and pigmentation intensity. At 24 h after laser irradiation, the level of IL-1β expression was significantly increased at laser powers higher than 1000 mW in the weak pigmentation group (*P* = 0.018, < 0.001, for 1000, 1500 mW, respectively by Dunnet’s multiple comparisons test), 1500 mW in the moderate pigmentation group (*P* = 0.005, by Dunnet’s multiple comparisons test), and higher than 700 mW in the strong pigmentation group (*P* = 0.003, < 0.001, < 0.001, for 700, 1000, 1500 mW, respectively by Dunnet’s multiple comparisons test). Taken together, these results suggested that stronger pigmentation was associated with greater induction of IL-1α/β expression for a given laser power. The levels of IL-1α/β mRNA expression increased according to the laser power in cells with weak and moderate pigmentation, but in the strong pigmentation group, the mRNA level was highest at 1000 mW.

### Evaluation of MMP/TIMP mRNA and protein expression levels

**Figure 4:**
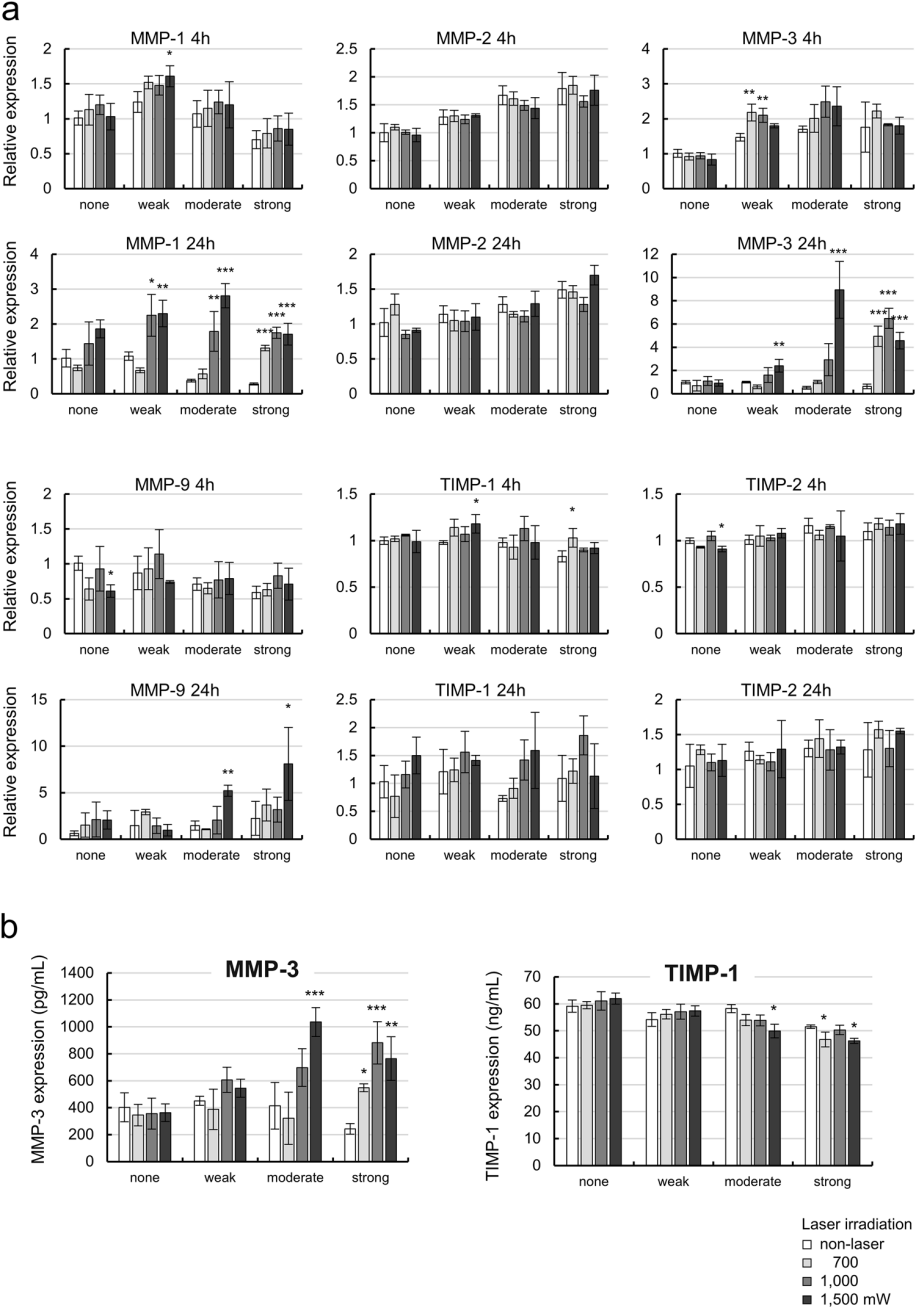
Expression levels of MMPs/TIMPs after 4 and 24 h of MP-laser irradiation of cells with each pigmentation intensity. (**a**) mRNA levels of MMPs/TIMPs after 4 and 24 h of MP-laser irradiation was measured by qPCR (n = 3). (**b**) MMP-3 and TIMP-1 protein expression in medium were measured by ELISA (n = 3). Data are represented as mean ± standard deviation of the mean. *P < 0.05, **P < 0.01, ***P < 0.001 by Dunnet’s multiple comparisons test.

The mRNA levels of MMPs/TIMPs were evaluated because it has been reported that IL-1α/β may induce MMPs in TM cells (Fig. 4a)^24–26^. In non-irradiated cells at 4 h, MMPs mRNA levels were slightly changed, but the group differences were not significant. The MMPs mRNA levels showed no changes due to MP laser irradiation at 4 h.

At 24 h, the MMP-1 mRNA level was significantly increased in cells irradiated at powers higher than 1000 mW in the weak (*P* = 0.010, = 0.008, for 1000, 1500 mW, respectively by Dunnet’s multiple comparisons test) and moderate (*P* = 0.002, < 0.001, for 1000, 1500 mW, respectively by Dunnet’s multiple comparisons test) pigmentation groups, and powers higher than 700 mW in the strong pigmentation group (*P* < 0.001, < 0.001, < 0.001, for 700, 1000, 1500 mW, respectively by Dunnet’s multiple comparisons test). MMP-3 mRNA was significantly increased in the weak and moderate pigmentation groups following irradiation at 1500 mW (*P* = 0.009, < 0.001, for weak, moderate, respectively by Dunnet’s multiple comparisons test). In the strong pigmentation group, MMP-3 mRNA was significantly increased following irradiation at powers of at least 700 mW (*P* < 0.001, < 0.001, < 0.001, for 700, 1000, 1500 mW, respectively by Dunnet’s multiple comparisons test). The levels of MMP-9 mRNA were significantly increased in the moderate and strong pigmentation groups following irradiation at 1500 mW (*P* = 0.001, = 0.045, for moderate, strong, respectively by Dunnet’s multiple comparisons test). The laser power required to induce MMP-1, -3, and -9 mRNA expression was lower in cells with higher pigmentation intensity, similar to IL-1α/β. In contrast, there were no changes in the mRNA levels of TIMP-1 and -2, which are endogenous inhibitors of MMPs, at 4 or 24 h after laser irradiation.

We observed significant upregulation of MMP-3 after MP laser irradiation, while TIMPs remained stable; the balance between MMPs and TIMPs has been suggested to play a role in ECM turnover in TM cells. Thus, we next evaluated the protein expression levels of MMP-3 and TIMP-1 after MP laser irradiation (Fig. 4b, Supplementary Table S2). The MMP-3 protein concentration in the culture medium was significantly increased in cells with high pigmentation intensity (*P* < 0.001, for 1500 mW in the moderate, and *P* = 0.021, < 0.001, = 0.011 for 700, 1000, 1500 mW, in the strong pigmentation respectively by Dunnet’s multiple comparisons test), consistent with the results of RT-qPCR. In addition, TIMP-1 showed slightly different basal protein expression levels in non-irradiated cells according to pigmentation intensity, but did not change with laser irradiation in nonpigmented cells or those with weak pigmentation. However, laser irradiation at 1500 mW in the moderate pigmentation group, and 700 or 1500 mW in the strong pigmentation group, resulted in significant decreases in the protein level of TIMP-1 (*P* = 0.011, = 0.042, = 0.027, for 1500 mW in the moderate, 700, 1500 mW in the strong, respectively by Dunnet’s multiple comparisons test). Although these results were not consistent with those of RT-qPCR, decreases in TIMP-1 according to pigmentation intensity and laser power were suggested. Taken together, these results suggested the upregulation of MMP-1, -3, and -9 expression, while TIMP-1 expression remained stable or decreased. Thus, MP laser irradiation regulated the MMP/TIMP ratio, which may promote ECM turnover.

### Evaluation of long-term MMP-3 protein levels

**Figure 5:**
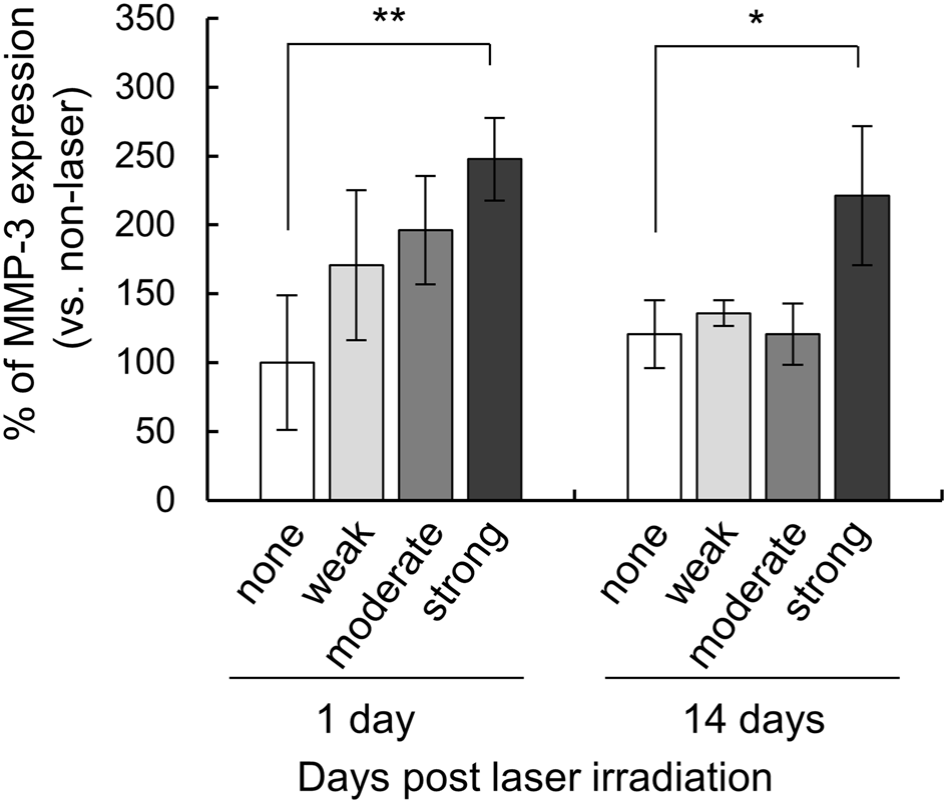
Long-term protein expression of MMP-3 after MP-laser irradiation. Protein levels of MMP-3 in the culture medium were measured by ELISA 1 or 14 days after laser irradiation (n = 3). Data are represented as mean ± standard deviation of the mean. *P < 0.05, **P < 0.01, ***P < 0.001 by Dunnet’s multiple comparisons test.

TM cells of each pigmentation intensity were irradiated with the MP laser at 1000 mW and the culture supernatants were sampled after 14 days. Medium exchange was performed 3 days prior to sampling. The MMP-3 expression levels (relative to non-irradiated controls) in the culture supernatant after laser irradiation of cells of each pigmentation intensity were calculated. The relative expression was calculated in the same way as for the 24-h ELISA results shown in Fig. 4b (Fig. 5, Supplementary Table S2). One day after laser irradiation, the strong pigmentation group showed a significantly greater increase in MMP-3 expression than the no-pigmentation group (*P* = 0.009, by Dunnet’s multiple comparisons test), even after 14 days (*P* = 0.011, by Dunnet’s multiple comparisons test). This suggested that MMP-3 expression was upregulated at least until 14 days after MP laser irradiation. The weak and moderate pigmentation groups showed an upward trend after 24 h, but after 14 days the expression levels of MMP-3 were comparable to the no-pigmentation group.

### Evaluation of αSMA, fibronectin, and collagen type I expression levels

**Figure 6:**
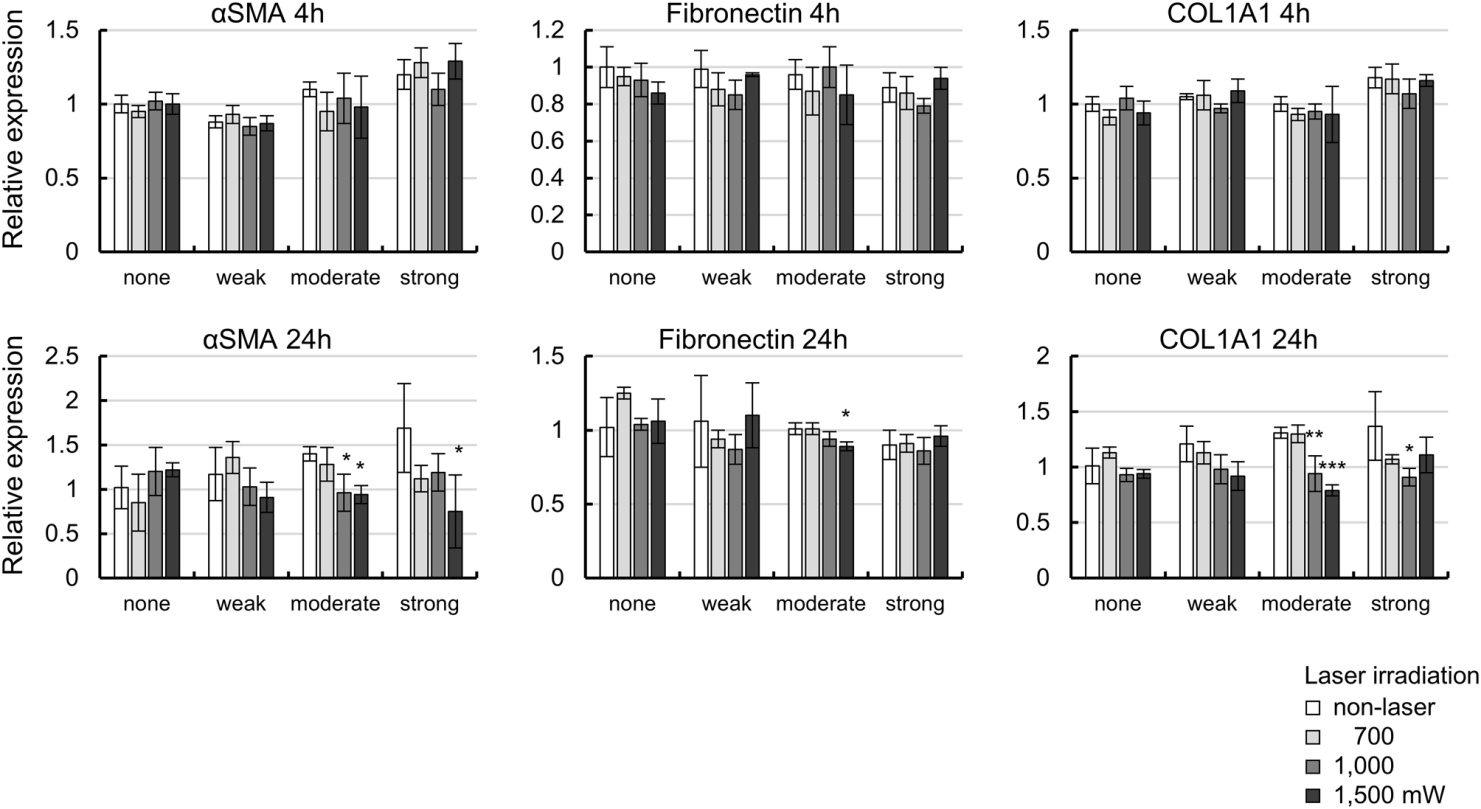
Expression levels of extracellular matrixes after 4 or 24 h of MP-laser irradiation of cells with each pigmentation intensity. mRNA levels of ECMs were measured by qPCR (n = 3). Data are represented as mean ± standard deviation of the mean (n = 3). *P < 0.05, **P < 0.01, ***P < 0.001 by Dunnet’s multiple comparisons test.

As MP laser irradiation was suggested to promote ECM turnover, the levels of αSMA, fibronectin, and COL1A1 expression, as markers of fibrosis and the ECM, were evaluated by RT-qPCR (Fig. 6). The levels of αSMA mRNA did not change at 4 h after laser irradiation, but significant decreases were observed with laser irradiation in some of the cells in the moderate (*P* = 0.021, = 0.017, for 1000, 1500 mW, respectively by Dunnet’s multiple comparisons test) and strong (*P* = 0.027 by Dunnet’s multiple comparisons test) pigmentation groups at 24 h. These observations suggested that the laser irradiation did not cause fibrosis of the cells. Fibronectin and COL1A1 showed similar results to αSMA at 24 h.

### Immunostaining of the ECM

**Figure 7:**
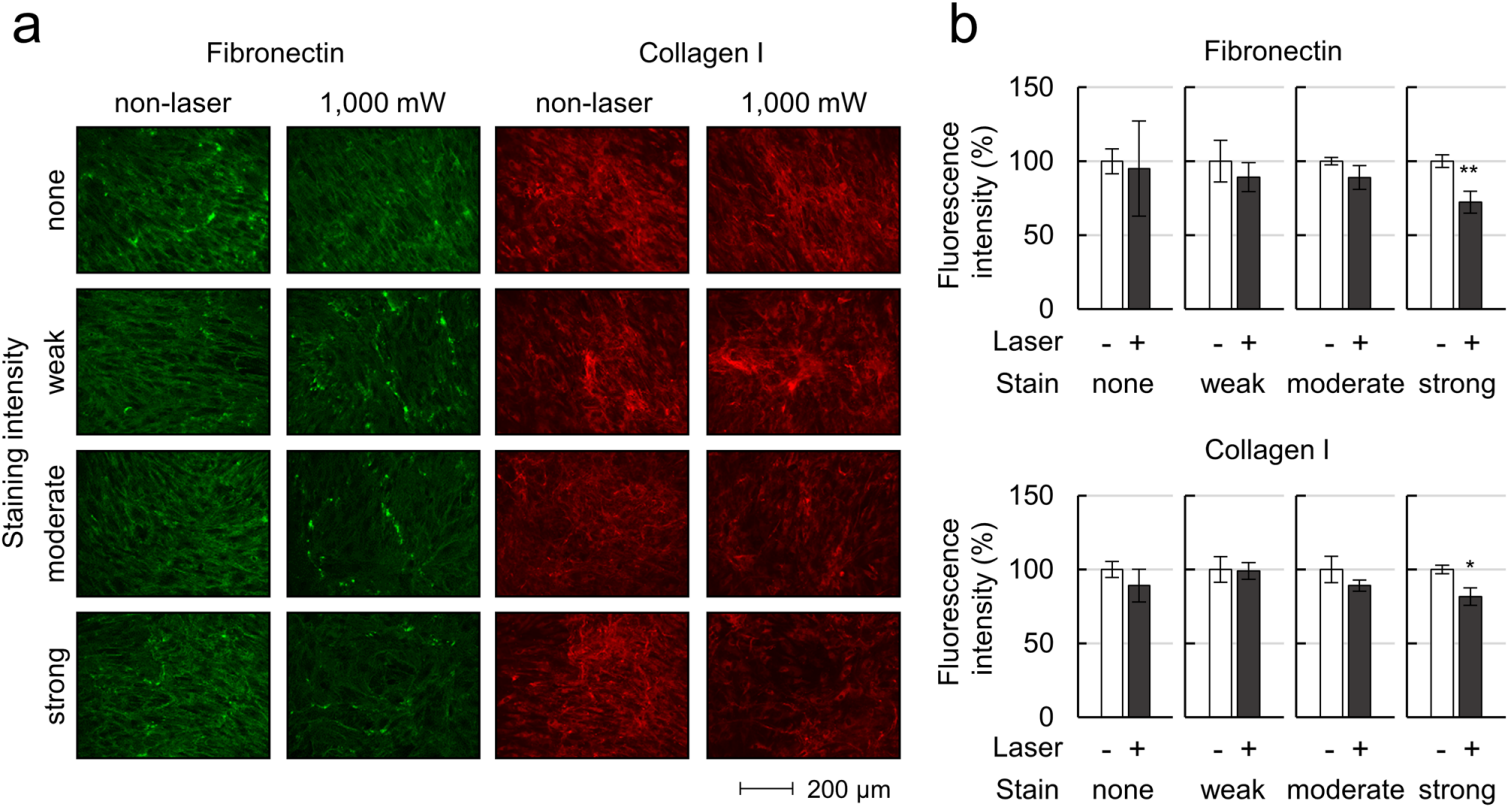
Changes in protein expression of extracellular matrixes 14 days after MP-laser irradiation of cells with each pigmentation intensity. (**a**) Immunocytochemistry of fibronectin and collagen I. Scale bars: 200 μm. (**b**) Fluorescence intensity of fibronectin and collagen I were calculated using Fiji Image-J (n = 3). Data are represented as mean ± standard deviation of the mean. *P < 0.05, **P < 0.01, ***P < 0.001 by student’s t-test.

As upregulation of MMP-3 expression was observed even 2 weeks after laser irradiation (Fig. 5), we also evaluated the protein levels of fibronectin and collagen type I in cells of each pigmentation intensity at 2 weeks after laser irradiation. A laser power of 1000 mW was selected based on the results of RT-qPCR. In the non-irradiated cells, there was no clear difference in the fluorescence intensity for fibronectin or collagen type I according to pigmentation intensity (Fig. 7a). For quantitative evaluation, the fluorescence intensity was compared between cells with and without laser irradiation (Fig. 7b). In cells with strong pigmentation, the fluorescence intensities of staining for fibronectin and collagen I after laser irradiation were 27.7% and 18.4% lower than those without laser irradiation, respectively (*P* < 0.05).

## Discussion

In this study, we performed MP laser irradiation of TM cells with different intensities of pigmentation, and demonstrated a relation between the pigmentation intensity of the TM and therapeutic effects of MLT. To our knowledge, this is the first study to show that MP laser irradiation leads to upregulation of cytokine expression, including of IL-1α/β, and subsequently of MMPs in cultured TM cells. We also showed that ECM-related protein levels were decreased after MP laser irradiation. Control TM cells without pigmentation showed no response to laser irradiation, consistent with previous in vitro studies of SLT^27^. Therefore, it was suggested that pigment is essential for the effects of MP laser irradiation.

The turnover and remodeling of the ECM of the TM are regulated by substrate-specific MMPs, such as interstitial collagenase (MMP-1), stromelysin-1 (MMP-3), gelatinases A (MMP-2) and B (MMP-9), as well as TIMPs^20,21,23^. It has been reported that MMP-3, -9, and TIMP-1 secretions are induced by IL-1α, IL-1β, and TNF-α in TM cells, and the MMP/TIMP ratio is involved in ECM turnover^22,24–27^. As shown in Fig. 3, MP laser irradiation caused significant increases in IL-1α/β mRNA expression from as early as 4 h after irradiation, while significant increases in the expression of MMP-1, -3, and -9 were observed at 24 h after irradiation. These observations suggest that MP laser irradiation upregulates the expression of these cytokines, and that IL-1α/β upregulation induces the expression of MMPs, consistent with previous reports. This has been reported previously both in vitro and in vivo as a possible mechanism underlying the responses to ALT and SLT, and is consistent with the results of the present study for MLT^37,38^. In this study, the expression of TIMPs did not change significantly after MP laser irradiation, which will alter the balance between MMPs and TIMPs (and thereby promote ECM turnover), resulting in the digestion of the ECM. The elevated expression of MMPs was also affected by the laser power (Fig. 4). Taken together, these results suggest that one of the mechanisms by which MLT lowers IOP is irradiation-induced upregulation of MMPs, including MMP-3.

There have been a number of reports regarding the correlation between the intensity of TM pigmentation and treatment effect of laser trabeculoplasty, but definitive conclusions have yet to be reached. For example, a modest positive correlation between the intensity of TM pigmentation and therapeutic effect of SLT was reported, while IOP reduction exceeding 20% was reported only in association with strong pigmentation^29,39^. Other studies suggested that the pigmentation intensity of the anterior angle of the eye was not related to the effect of SLT treatment^40–42^. With regard to MLT, there has been only one clinical study comparing the success rates of SLT and MLT, which showed no significant correlation between pigmentation intensity and the success rate of either method^17^. However, in the present study, MP laser irradiation significantly upregulated the expression of IL-1α/β and MMPs in TM cells with strong pigmentation, while no significant upregulation was observed in cells with weak pigmentation (Figs. 3 and 4). Our ELISA results also supported those of RT-qPCR and immunohistochemistry, and significant upregulation of MMP-3 in the medium was confirmed only in TM cells with strong pigmentation (Figs. 4b and 5). It is reasonable to speculate that the upregulated and secreted MMP-3 can affect the surrounding tissues, including non-pigmented and pigmented TM cells, juxtacanalicular tissues, and Schlemm’s canal, in a paracrine manner, resulting in decreased ECM deposition in the tissue. The upregulation of MMPs was affected by the intensity of pigmentation, suggesting that TM with pigmentation may be more likely to benefit from MLT. However, caution is required because IL-1α/β and MMP upregulation was suppressed in TM cells with strong pigmentation under MP laser irradiation at 1500 mW compared to 1000 mW. Because the results of CCK-8 assay showed the cytotoxicity by irradiating cells with strong pigmentation intensity at 1000, 1500 mW (Fig. 2b), it is possible that the cellular response can be weakened when the cells absorb an excessive amount of energy. Further study will be needed on more detailed cytotoxicity assessment and its contribution to the attenuation of cellular responses. In clinical practice, it is possible that more efficient IOP reduction may be achieved by adopting the optimal MP laser irradiation power according to the intensity of TM pigmentation in each individual patient.

As MP laser irradiation was associated with increases in MMP mRNA and MMP-3 protein levels, we evaluated the ECM protein level by immunostaining. Laser irradiation of TM cells with strong pigmentation at 1000 mW resulted in a significant decrease in the fluorescence intensity of fibronectin compared to non-irradiated controls (Fig. 7). However, there was no significant correlation between the ECM protein level and pigmentation intensity. The slight decrease in ECM protein level observed in the present study may have occurred because MP laser irradiation was performed on normal cultured TM cells, and not in a disease model with elevated ECM expression. It has been reported that remodeling of the ECM structure enhances the aqueous humor outflow pathway, especially in the case of TM outflow with glaucomatous change^13,43^. Therefore, it is possible that MMPs digest ECM with an abnormal structure and create a new one with a different structure. Further studies using glaucoma models with elevated ECM expression are required to investigate this issue.

This study had some limitations. First, the three levels of pigmentation of cultured TM cells were not correlated with the pigmentation intensity grade of human TM in clinical practice. In this study, weak/moderate/strong intensity of pigmentation were determined only visually by authors to be with in the clinical grading, but because clinical grading is largely subjective, quantitative methods of measurement are being studied^44–46^. These studies will facilitate future detailed in vitro studies of the relationship between pigmentation intensity and treatment efficacy. Second, in this study, monolayer cultures of artificially pigmented TM cells were subjected to laser irradiation, so the absorption of energy may have been different from that in TM tissue in vivo, although we measured the absorbed power using a power meter. Previous studies in human TM tissues and rabbit TM in vivo have demonstrated that laser irradiation induces the expression of chemokines (including IL-8), which recruit monocytes^47,48^. As we focused only on autocrine-like effects on TM cells in this study, further in vivo studies are needed to assess the effects of pigmentation concentration and the immunological response in more detail. In addition, the elevations of baseline expressions of IL1α/β and some MMPs induced by uptake of melanin granules on the cells have not been eliminated in the present study, although these effects were considered limited compared to the laser-induced effects. Moreover, we used immunostaining to evaluate the changes of protein expressions in the present study because it was difficult to quantify proteins by quantitative way such as western blotting due to the influence of artificially introduced melanin pigment granules. At the same time, comparison of culture on plates and laser irradiation of tissue has not been performed in the present study. Detailed studies will be needed in the future. Finally, in accordance with previous reports, it is thought that elevated IL-1α/β caused the increase in MMPs, but a detailed study by blocking the pathway has not been conducted in the present study. We would like to perform the evaluation of the pathway using ROS, IL-1 and other inhibitors in the future study.

In summary, MP laser irradiation was shown to upregulate the expression of IL-1α/β and MMPs in pigmented cultured TM cells, which may partially explain the mechanism underlying the IOP-lowering effect of MLT. The pigmentation intensity of TM cells was also shown to be significantly related to the response to MP laser irradiation and upregulation of MMP expression. Therefore, it may be possible to improve the efficacy of MLT by adjusting the laser irradiation settings according to the pigmentation intensity of the TM tissue in individual patients.

## Supplementary Information

Supplementary Figures.

Supplementary Table S1.

Supplementary Table S2.

## Data Availability

All data generated or analysed during the current study available from the corresponding author on reasonable request.
