# Supplementary material for: Effect of pigmentation intensity of trabecular meshwork cells on mechanisms of micropulse laser trabeculoplasty
Source: Sci Rep. 2022 Jun 22;12:10535. doi: 10.1038/s41598-022-14637-5 (PMC9217947; doi:10.1038/s41598-022-14637-5)
Supplement: Supplementary file 1 — Supplementary Figures. [file 41598_2022_14637_MOESM1_ESM.pdf]

Effect of pigmentation intensity of trabecular meshwork cells on mechanisms of micropulse laser trabeculoplasty

Shota Shimizu, Megumi Honjo, Koichiro Sugimoto, Michiaki Okamoto, Makoto Aihara.

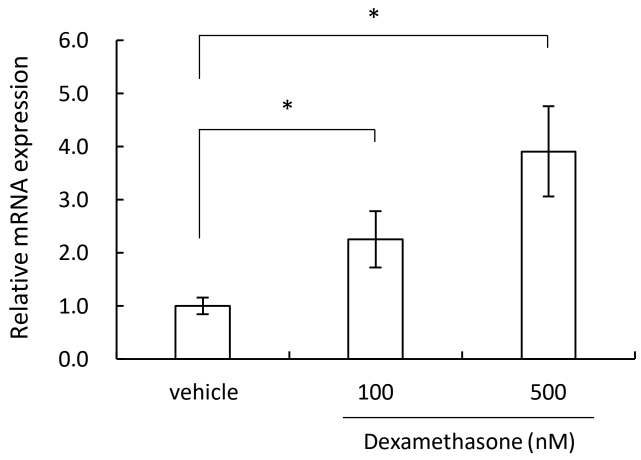

Supplementary Figure S1 Dexamethasone-induced upregulation of MYOC (n = 4). Data are represented as mean ± standard deviation of the mean. *P < 0.05, **P < 0.01, ***P < 0.001 by Dunnet’s multiple comparisons test.

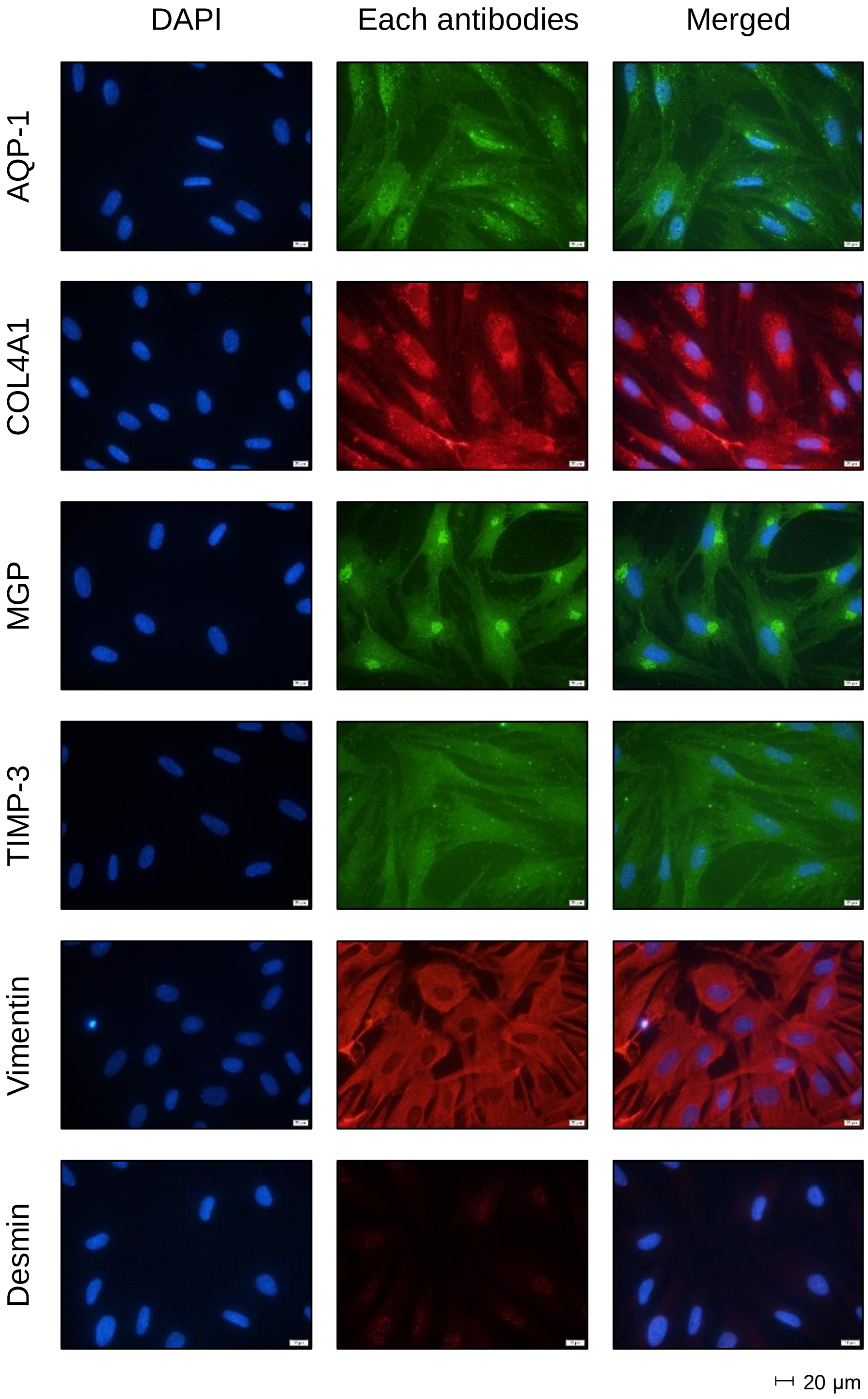

Supplementary Figure S2 Characterization of hTM cells. The hTM cells used in the study were positive for AQP-1, COL4A1, MGP, TIMP-3, and vimentin, and were negative for desmin.
