# Supplementary material for: Effect of pigmentation intensity of trabecular meshwork cells on mechanisms of micropulse laser trabeculoplasty
Source: Sci Rep. 2022 Jun 22;12:10535. doi: 10.1038/s41598-022-14637-5 (PMC9217947; doi:10.1038/s41598-022-14637-5)
Supplement: Supplementary file 2 — Supplementary Table S1. [file 41598_2022_14637_MOESM2_ESM.pdf]

Effect of pigmentation intensity of trabecular meshwork cells on mechanisms of micropulse laser trabeculoplasty

Shota Shimizu, Megumi Honjo, Koichiro Sugimoto, Michiaki Okamoto, Makoto Aihara.

Supplementary Table S1 Primer sequences

	**Forward (5'-3')**	**Reverse (5'-3')**
GAPDH	AATTCCATGGCTCCGTCAAG	ATCGCCCCACTTGATTTTGG
IL-1α	TGTATGTGACTGCCCAAGATGAAG	AGAGGAGGTTGGTCTCACTACC
IL-1β	ATGATGGCTTATTACAGTGGCAA	GTCGGAGATTCGTAGCTGGA
MMP-1	GCTTACGAATTTGCCGACAGAG	TTCCTCAGAAAGAGCAGCATCG
MMP-2	ATAACCTGGATGCCGTCGT	AGGCACCCTTGAAGAAGTAGC
MMP-3	CACTCACAGACCTGACTCGGTT	AAGCAGGATCACAGTTGGCTGG
MMP-9	ACGATGACGAGTTGTGGTCC	CGGAGTAGGATTGGCCTTGG
TIMP-1	TGCGGATACTTCCACAGGTC	GCAGTTTGCAGGGGATGGATA
TIMP-2	ATGCACATCACCCTCTGTGA	CTCTGTGACCCAGTCCATCC
Fibronectin	AAGCAAGCCCGGTTGTTATG	AAACCAACGCATTGCCTAGG
COL1A1	CAGCCGCTTCACCTACAGC	TTTTGTATTCAATCACTGTCTTG
αSMA	TTGTCCACCGCAAATGCTTC	AGGTAACGAGTCAGAGCTTTGG
